# Brainstem Correlates of a Cold Pressor Test Measured by Ultra-High Field fMRI

**DOI:** 10.3389/fnins.2020.00039

**Published:** 2020-01-31

**Authors:** Mariëlle C. Hendriks-Balk, Fatma Megdiche, Laura Pezzi, Olivier Reynaud, Sandra Da Costa, Domenica Bueti, Dimitri Van De Ville, Grégoire Wuerzner

**Affiliations:** ^1^Service of Nephrology and Hypertension, Department of Medicine, Lausanne University Hospital and University of Lausanne, Lausanne, Switzerland; ^2^Centre d’Imagerie BioMédicale (CIBM), Ecole Polytechnique Fédérale de Lausanne, Lausanne, Switzerland; ^3^Faculty of Medicine, University of Geneva, Geneva, Switzerland; ^4^Medical Image Processing Laboratory, Ecole Polytechnique Fédérale de Lausanne (EPFL), Lausanne, Switzerland

**Keywords:** cold pressor test, brainstem, BOLD, fMRI, blood pressure, sympathetic nervous system

## Abstract

**Introduction:**

Modern imaging techniques such as blood oxygen level-dependent (BOLD) functional magnetic resonance imaging (fMRI) allow the non-invasive and indirect measurement of brain activity. Whether changes in signal intensity can be detected in small brainstem regions during a cold pressor test (CPT) has not been explored thoroughly. The aim of this study was to measure whole brain and brainstem BOLD signal intensity changes in response to a modified CPT.

**Methods:**

BOLD fMRI was measured in healthy normotensive participants during a randomized crossover study (modified CPT vs. control test) using ultra-high field 7 Tesla MRI scanner. Data were analyzed using Statistical Parametric Mapping (SPM) in a whole-brain approach, and with a brainstem-specific analysis using the spatially unbiased infra-tentorial template (SUIT) toolbox. Blood pressure (BP) and hormonal responses (norepinephrine and epinephrine levels) were also measured. Paired *t*-test statistics were used to compare conditions.

**Results:**

Eleven participants (six women, mean age 28 ± 8.9 years) were analyzed. Mean arterial BP increased from 83 ± 12 mm Hg to 87 ± 12 mm Hg (*p* = 0.0009) during the CPT. Whole-brain analysis revealed significant activations linked to the CPT in the right supplementary motor cortex, midcingulate (bilateral) and the right anterior insular cortex. The brainstem-specific analysis showed significant activations in the dorsal medulla.

**Conclusion:**

Changes in BOLD fMRI signal intensity in brainstem regions during a CPT can be detected, and show an increased response during a cold stress in healthy volunteers. Consequently, BOLD fMRI at 7T is a promising tool to explore and acquire new insights in the comprehension of neurogenic hypertension.

## Introduction

Increased sympathetic tone is associated with the initiation and maintenance of many forms of hypertension (Esler and Kaye, 2000; Guyenet, 2006; Fisher and Paton, 2012), and there is growing evidence that the sympathetic tone is regulated by a network of neuronal populations in the brainstem, the spinal cord and the hypothalamus (Sved et al., 2003; Guyenet, 2006). Several crucial areas in the medulla in the brainstem contribute to the cardiovascular control, such as the rostral ventrolateral medullar (RVLM), the caudal ventrolateral medulla (CVLM) and the nucleus of the solitary tract (NTS) (Guyenet, 2006). Together these areas play key roles in the baroreceptor reflex, which is a powerful negative feedback reflex to control cardiovascular function, in addition to having specific, independent contributions to the cardiovascular control (Sved et al., 2000, 2003). Careful physiological investigations in animals show that the baroreceptor sensory nerves from the carotid bodies and the aortic arch project to the NTS resulting in an increased activity of the NTS neurons in response to increases in blood pressure. These excitatory neurons of the NTS increase the activity of the CVLM of which the inhibitory neurons project to the RVLM. The increased activity of the inhibitory neurons causes an inhibition of the tonically active sympathoexcitatory neurons in the RVLM and hence a decrease in sympathetic outflow to blood vessels (Sved et al., 2003; Dampney, 2016).

Imaging studies using functional magnetic resonance imaging (fMRI) have shown that some cortical brain regions (e.g., the insular and cingular cortex) are involved in cardiovascular control when using physiological manipulations that increase the sympathetic nervous system response, such as the cold pressor test (CPT), hand grip exercise, the Valsalva maneuver and lower body negative pressure (Macefield and Henderson, 2016). However, most of these fMRI studies did not explore specifically the brainstem region.

Due to the relative small size of the brainstem nuclei compared to cortical structures, its functional heterogeneity, and the close proximity of cerebral vessels, fMRI of the brainstem region in humans is challenging (Brooks et al., 2013). In the last two decades, significant advances in brainstem fMRI image acquisition and processing resulted in improved accuracy and feasibility of fMRI in the brainstem region (Beissner, 2015; Sclocco et al., 2018). Only a few studies using blood-oxygen-level-dependant (BOLD) fMRI focusing on the brainstem regions have been conducted in humans. Their objectives were to couple muscle sympathetic nervous activity to BOLD signal intensities in the brainstem of awake human subjects and to compare the brainstem response with patients with obstructive sleep apnea (Macefield and Henderson, 2010; Lundblad et al., 2014) or to measure brain response to specific tasks such as static handgrip (Sander et al., 2010), isometric forearm contraction (Coulson et al., 2015) or a Stroop test (Naumczyk et al., 2017) or a response to a pharmacological test (Gerlach et al., 2019). The objective of our study was to measure BOLD fMRI in response to a modified CPT in the brainstem using ultra-high field imaging at 7 Tesla, and to evaluate the added value of a brainstem-specific analysis compared to a whole brain analysis, in healthy volunteers.

## Materials and Methods

### Study Population

Healthy, normotensive, participants without any drug abuse and/or consumption and aged over 18 years were eligible to participate in the study. None of the subjects had any contraindications for MRI, or was pregnant. Prior to initiation of any study-related procedures, written informed consent was obtained from every participant. Participants unable to understand written French were excluded.

### Study Design/Procedure

This was a randomized single center crossover study (modified CPT vs control stimulation). On the study day, participants arrived at the MRI facility (Centre d’Imagerie BioMédicale, CIBM, EPFL, Lausanne) and were installed in a quiet study room outside the MRI room. A catheter was inserted in one arm to allow blood samples to be taken. After 10 min rest in the supine position, ice-cold gel packs (−2 to 4°C at the site of contact) were placed around both feet by the investigators for 2 min (Supplementary Figure S1). Blood samples were taken after 10 min baseline conditions and after 2 min of the CPT. Mean systolic blood pressure (BP), diastolic BP and pulse frequency at rest (10 measurements) and during the CPT (2 measurements) were measured at an interval rate of 1 min using a MAGLIFE C Plus monitor (Schiller Medical S.A.S., France). Mean arterial BP was calculated as 1/3 systolic BP + 2/3 diastolic BP.

**FIGURE 1:**
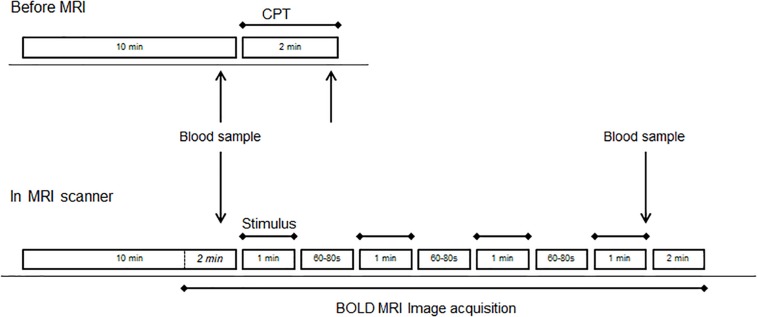
Study design before (top drawing) and during MRI (bottom drawing). Each of the four runs consisted of four blocks of the same stimulus (CPT or control). The order of runs with CPT (stress) or blanket (control) was randomized across subjects.

Participants were then transferred to the MRI scanner. After 10 min of “rest” during which structural images were acquired, the “active” fMRI run started with 2 min of functional image acquisition, during which a blood sample was taken, followed by four stimulations of 1 min separated by 60–80 s of rest. After the fourth stimulation, a blood sample was taken and image acquisition continued for another 2 min (see Figure 1).

For the modified CPT during MRI sessions, ice-cold gel packs (−2 to 4°C at the site of contact) were put around both feet by the investigators, while a blanket was used as a control stimulus during the control runs to allow for the differentiation between a sensation of touch and the perception of cold stimulus (Supplementary Figure S1). fMRI acquisitions were done twice with the stressor (CPT) and twice with a control (blanket), in a randomized order across subjects. The participants were instructed not to move and to breathe normally during the MRI sessions. Respiration patterns and the heart rates were monitored during fMRI with a plethysmograph belt around the chest and a pulse oximeter on the tip of the index finger, which were provided by the MRI scanner vendor. Finally, plasma norepinephrine and epinephrine levels were measured in the blood samples using liquid chromatography tandem mass spectrometry (Dunand et al., 2013).

### MRI Data Acquisition

Subjects were laying in a supine position inside a 7 Tesla/680 mm head-only MRI scanner (Siemens, Germany) equipped with a head gradient insert (80 mT/m maximum gradient strength, 333 T/m/s maximum slew-rate) and a 32-channel RF-head coil (Nova Medical Inc., MA, United States). The respiration pattern and cardiac rate were simultaneously monitored while subjects were in the scanner. Physiological data were recorded using a Physiological Monitoring Unit (Siemens) and used for retrospective physiological noise correction during the fMRI data analysis. Structural images were acquired using the T1-weighted high resolution MP2RAGE sequence (Marques et al., 2010) with the following parameters: matrix size = 256 × 240 × 160, resolution = 1 mm × 1 mm × 1 mm, TR/TE/TI1/TI2 = 5500 ms/1.87 ms/750 ms/2350 ms (total scan time of 10 min). The field of view covered the entire sensitive volume of the RF-head coil.

Functional magnetic resonance imaging was achieved with a 3D-EPI sequence using advanced accelerated BOLD fMRI techniques (Narsude et al., 2016; Reynaud et al., 2017), with the following parameters: TR/TE = 2000/26 ms, Field-of-view: 200 × 200 × 160, matrix size 100 × 100 × 80, spatial resolution 2 mm × 2 mm × 2 mm, 314 whole-brain volumes (total scan time 10 min 33 s per run). To enhance the temporal resolution, we used a 4-fold acceleration factor using GRAPPA 2 × 2 along the two phase encoding directions PE and PE2, and control aliasing along PE2 (Narsude et al., 2016; Reynaud et al., 2017). And to maximize the SNR, the sequence flip angle was adapted to the repetition times between two consecutive echo trains in 3D imaging (Ernst angle = 13°).

### fMRI Data Analysis

Individual data were inspected for the linear and rotational motion parameters. Functional MRI data of one male subject and of two runs (one control and one stressor) of one female subject were excluded due to insufficient quality (e.g., geometric distortions of the images, presence of secondary images).

The individual functional images were preprocessed as described below using Statistical Parametric Mapping (SPM12) software (Wellcome Department of Imaging Neuroscience, London, United Kingdom)^1^ implemented in MATLAB (Mathworks Inc., MA, United States).

#### Whole Brain Analysis

The functional images of each run were realigned, co-registered to the T1-weighted image, spatially normalized to a standard brain atlas [Montreal Neurological Institute (MNI) template space] and smoothed (isotropic Gaussian kernel with 4 mm full width at half-maximum).

A first level general linear model was fitted to the subject’s preprocessed data to estimate the parameters for each run. Each run with the four blocks of stimulation was modeled as four boxcars of 1-minute duration and convolved with the canonical hemodynamic response function (basic model fit). After each stimulus, a decay period of 14 s was added to the model. The model also included the six motion correction parameters for head movement, physiological correction parameters [cardiac and respiratory behavior but not heart rate using RETROICOR (Glover et al., 2000)], and signal drift as effects of no interest (co-regressors) in the multiple regression analysis (Reynaud et al., 2017; Supplementary Figure S2). As the CPT might result in an important increase in heart rate, we did not correct for the heart rate in the model.

A high pass filter with cutoff frequency 1/300 Hz was used. For each subject, a t-contrast between cold stress and control was computed. These single-subject contrast images were then used in the second level analysis (group level). The statistical threshold for the *t*-test was set to *p* < 0.001, uncorrected for multiple comparison, due to the small number of subjects.

Voxels that exhibited significant differences were color-coded and displayed over the MNI reference image using the xjView toolbox^2^. Significant clusters were identified using the xjView and the Automated Anatomical Labeling toolboxes. Regions of interest were created from the significant clusters and analyzed using the MarsBaR toolbox within SPM. For each subject, the average BOLD signal across all voxels at each time point of a given ROI was extracted. These raw time courses per subject were normalized to the average BOLD signal at the onset of the stimulation and then averaged across repetitions and conditions. Group averages of time courses during the stimulation were extracted and plotted for each ROI.

#### Brainstem-Specific Analysis

A brainstem-specific analysis was performed using a high-resolution atlas template specific for the brainstem and cerebellum from the SUIT toolbox (Diedrichsen, 2006) within SPM. The first run (stress and control) was used for the brainstem-specific analysis because the brainstem region is more sensitive to motion and because movement increased with time. After realignment and co-registration, the brainstem and cerebellum were isolated from each subject’s structural image. Using the SUIT atlas, the images were spatially normalized into MNI space, and together with the functional images resliced and smoothed (isotropic Gaussian kernel with 4mm full width at half-maximum). Significant signal intensity changes were determined using an identical model as the one used for the whole brain analysis. Voxels that exhibited significant differences were color-coded and displayed over the reference SUIT image. Significant clusters were identified, then used to create ROIs and for the extraction of the BOLD responses as described above for the whole brain analysis.

### Statistics

Statistical analyses were performed using STATA 14.0 (StataCorp, College Station, TX, United States). Data were represented as the mean ± standard deviation (SD). A paired *t*-test was used to test for significant differences between mean hemodynamic and hormonal variables during the CPT and baseline. Statistical analyses on the raw time courses of the BOLD signal changes were performed using a one-way ANOVA. A *p*-value of <0.05 was considered as statistically significant.

## Results

Twelve healthy subjects were included in the study. MRI data quality of one participant was insufficient to be analyzed and was not used in the study. Hence, the final study included data from the remaining eleven participants [six women, age 28 ± 8.9 years (mean ± SD)]. The study was well tolerated. None of the participants reported adverse effects or pain during or after the experiment.

### Blood Pressure and Hormonal Response

The systolic, mean and diastolic BP were significantly increased by the modified CPT (Table 1). The level of norepinephrine tended to increase upon the stress test, while the epinephrine level remained stable. No change in heart rate was detected during the CPT.

**TABLE 1:** Blood pressure (BP) (*n* = 10) and hormone levels (*n* = 8) before and during the cold pressor test.

	Baseline (mean ± SD)	CPT (mean ± SD)	*p*-value
Systolic BP (mmHg)	114 ± 15	118 ± 17	**0.0266**
Diastolic BP (mmHg)	68 ± 11	71 ± 10	**0.0019**
Mean arterial BP (mmHg)	83 ± 12	87 ± 12	**0.0009**
Norepinephrine (nM)	1.22 ± 0.67	1.36 ± 0.41	0.36
Epinephrine (nM)	0.14 ± 0.12	0.12 ± 0.09	0.37

*Bold values show that the difference between baseline and CPT is significant.*

### BOLD fMRI Signal Intensity Changes – Whole-Brain Analysis

**FIGURE 2:**
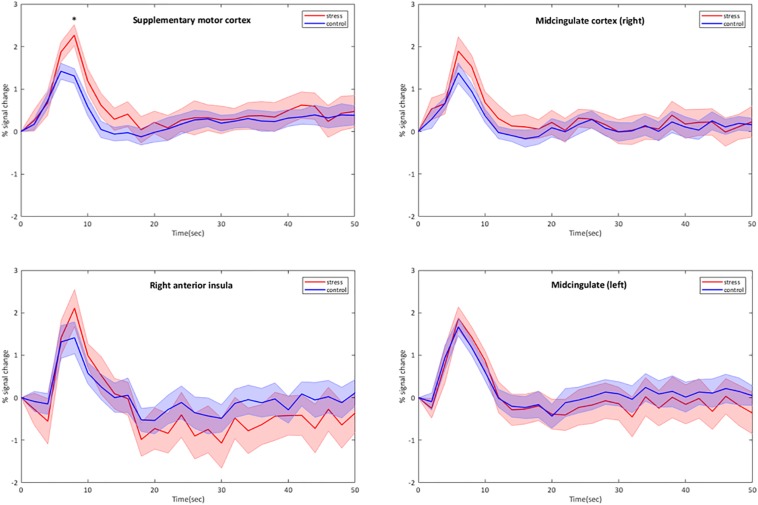
Group-averaged time courses of BOLD signal intensity changes (mean ± Standard Error) in cortical brain regions activated by the CPT. BOLD responses are plotted without correction for motion, physiological noise and scanner drift. The four stimulation blocks are averaged and normalized at subject level before averaging at group level (*n* = 11). ^∗^*p* < 0.05, CPT vs. control, one-way ANOVA.

Group results for the contrast CPT vs. control (blanket) of the BOLD fMRI data revealed significant activations in the supplementary motor cortex (in the region dedicated to the representation of the lower body), the midcingulate and the right anterior insular cortex (see Table 2 for the MNI cluster coordinates, *T*- and *Z*-scores and cluster sizes, and Supplementary Figure S3 for the projection on the brain surface). BOLD responses within these regions showed a brief increase, followed by a peak at 6–8 s after the stimulation, and a return to baseline after 12–15 s (Figure 2). This change in BOLD signal was significantly higher for CPT compared to the control stimulus in the supplementary motor cortex (2.27 ± 0.192 vs. 1.42 ± 0.159% signal change, *p* < 0.05). The same trend was seen in the right anterior insula and the midcingulate, but those signal increases did not reach statistical significance (Figure 2). Time courses of BOLD signal intensity changes are reported without any correction for physiological noise, motion and scanner drift. No significant BOLD signal intensity changes were seen within the brainstem region using the whole-brain analysis.

**TABLE 2:** Cluster coordinates, *T*- and *Z*-scores and cluster size for regions showing significant activations for CPT for a whole-brain analysis and a brainstem-specific analysis (*n* = 11; *p* < 0.001, uncorrected).

	x	y	z	*T*-score	*Z*-score	Cluster size
**Whole-brain analysis:**						
Right supplementary motor area	6	12	46	8.83	4.57	64
(Right) midcingulate	8	−12	42	6.85	4.08	12
Right anterior insula	46	18	−8	5.52	3.66	8
Left midcingulate	0	14	30	5.18	3.53	7
**Brainstem-specific analysis:**						
Medulla	−2	−44	−49	6.08	3.85	27

*Cluster coordinates are given in Montreal Neurological Institute (MNI) space.*

### BOLD fMRI Signal Intensity Changes – Brainstem-Specific Analysis

**FIGURE 3:**
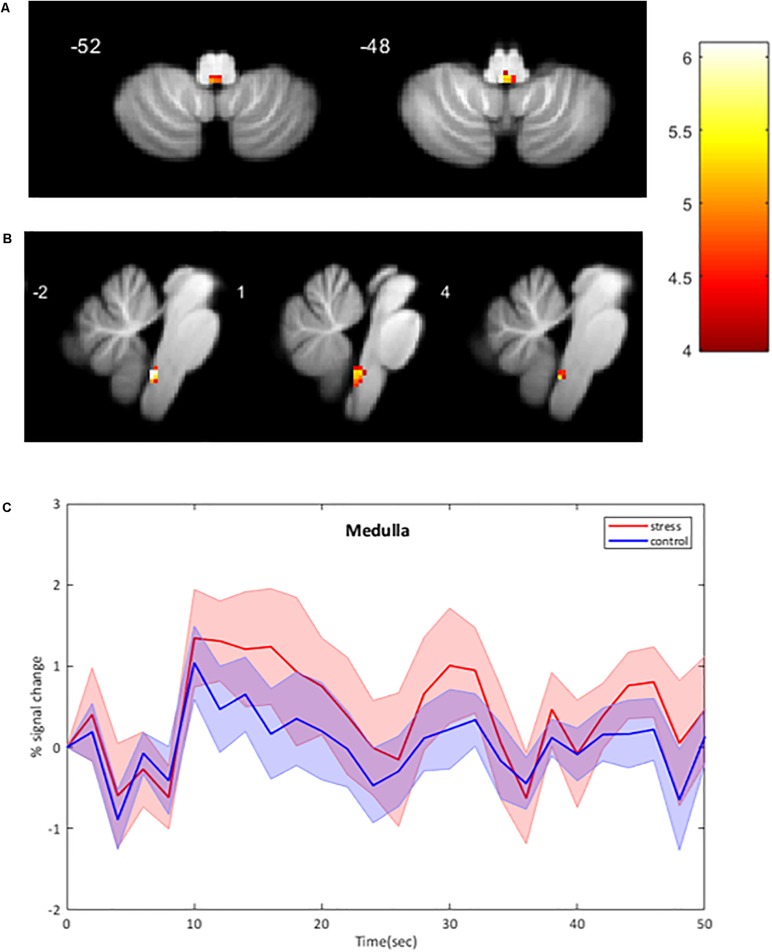
BOLD signal intensity changes in the human brainstem. **(A,B)** Significant changes at group level (*n* = 11) during CPT versus control overlaid onto the SUIT reference image. BOLD responses are corrected for motion, physiological noise and scanner drift. A: axial images; B: sagittal images. Threshold of 4.14 for *p* < 0.001 (uncorrected), cluster threshold of 6. *L* Left, *R* Right. **(C)** Group-averaged time course of BOLD signal intensity changes in the medulla (mean ± Standard Error) activated by CPT. BOLD responses are plotted without corrections for motion, cardiac and respiratory noise and scanner drift. The four stimulation blocks are averaged and normalized to the average BOLD signal at the onset of the stimulation at subject level before averaging at group level (*n* = 11). No significant differences between conditions.

Using the brainstem-specific analysis, one cluster of voxels in the medulla of the brainstem showed an increased BOLD response during CPT compared to control (see Figure 3 and Table 2 for the MNI cluster coordinates, *T*- and *Z*-scores and cluster size). This cluster lies in the dorsal part of the medulla where the NTS, the dorsal motor nucleus of the vagus nerve and some raphe nuclei are located. The peak BOLD response to stimulation was observed at 10 s with additional peaks for the CPT, while the response for the control stimulus returned to baseline after 20 s (see Figure 3). However, these differences between the two stimulations did not reach significance. As for the whole brain analysis, time courses of BOLD signal changes are reported without any correction for the physiological noise, movement artifacts and scanner drift.

## Discussion

This study shows that a modified CPT using gel packs leads to detectable BOLD signal changes in brain and brainstem regions related to stress. In particular, we found in healthy volunteers increases in the supplementary motor area of the lower body, the right insula and midcingulate cortex, as well as in the dorsal medulla where the NTS, the dorsal motor nucleus of the vagus nerve and some raphe nuclei are located. Furthermore, the analysis of the BOLD responses within the activated regions showed that the BOLD response peaked 6–10 s after the stimulation onset, and returned to baseline after 12–20 s.

Based on previous findings (Topolovec et al., 2004; Gerlach et al., 2019) in the brainstem, we believe that the cluster activated in the dorsal medulla might encompass the NTS and the dorsal motor nucleus of the vagus nerve, which are adjacent to each other. This is confirmed by using the mask for the NTS developed by Priovoulos et al. (2019).

The RVLM is likely to be implicated in the BP increase as a key control center for sympathetic nervous output. The absence of change in BOLD signal intensity in the RVLM and CVLM during the 0 to 60 s time range may be a result of a two steps excitatory and inhibitory component occurring within 1 min of stimulation. Indeed, according to Yamamoto et al. (1992), BP increases upon the CPT is in the first 30 s due to a slight increase in cardiac output while muscle sympathetic nerve activity does not change. In the time range 30–90 s of CPT the BP increase is due to increase in muscle sympathetic nerve activity without changes in cardiac output.

The observed rise in BP was smaller than shown in previous studies (Wood et al., 1984; Victor et al., 1987), which can be explained by the lower thermal conductivity of the ice-cold gel packs compared to the immersion in icy water. In addition, BP is measured intermittently at intervals of 1 min. This non-continuous measurement of BP may have missed the peak effect on BP. The absence of expected increase in heart rate may be explained by several reasons. First, the time at which blood pressure was measured (1 min interval) may have missed acute increase in heart rate within the first minute of stimulation. Secondly if the dorsal motor nucleus of the vagus nerve was indeed activated it may have affected the ventricular function more than the heart rate as was shown by Machhada et al. (2015, 2016). Finally, the low level of stimulation may have been insufficient to increase the heart rate.

The supplementary motor area is involved in controlling many aspects of motor behavior; e.g., motor preparation, selection of and learning action sequences, initiation of movement and inhibition and change of actions (Tanji, 1996; Nachev et al., 2008). Although we used a passive stress test, without movement of the participants, we measured an increase in BOLD signal intensity changes in the supplementary motor area of the lower body. A possible explanation is that the modified CPT might have implicitly activated a brain network involved in motor planning in order to facilitate and/or accelerate behavioral responses to the cold stress, in parallel of activating a mechanism to inhibit the execution of the planned responses if the motor action is not required or prohibited. A previous study showed that the perception of objects without any contact with them resulted in activation of the supplementary motor cortex (Grezes and Decety, 2002). Our findings suggest that the supplementary motor area might be activated in a similar way by the cold and touch sensation during the CPT in our study. Finally, the activation of the midcingulate cortex and the insula during the modified CPT is also in line with previous studies using cold temperature sensation on the foot, forehead or hand (Harper et al., 2000; Frankenstein et al., 2001; Fulbright et al., 2001; Richardson et al., 2013).

The increase of BOLD signal intensity in the medulla was shown by the brainstem-specific analysis and was not detectable using the whole-brain analysis, in contrast to previous studies that did detect BOLD signal changes in the brainstem using whole-brain analysis. A study in healthy adolescents showed an initial increase in the BOLD signal in the medulla during a right food CPT (Richardson et al., 2013), whereas another study in children showed a decrease of the BOLD signal in the same region during a forehead CPT (Macey et al., 2005). These studies also reported increases in the BOLD signal in midbrain and pontine regions during forehead CPT in children and healthy adults (Harper et al., 2003; Macey et al., 2005), whereas decreases of the BOLD signal in the pons was measured in healthy adolescents during a CPT of the right foot (Richardson et al., 2013). However, none of these studies identified the specific brainstem nuclei as they used relative large ROIs, which included either the dorsal part or the ventral part or both parts together of the medulla, the pons and/or the midbrain.

Changes in BOLD responses in the medulla of the brainstem were also reported in studies using other physiological tests, like the isometric forearm contraction (Coulson et al., 2015), a static handgrip exercise (Topolovec et al., 2004; Sander et al., 2010), a Valsalva maneuver and maximal inspiration (Topolovec et al., 2004) as well as in a study using a pharmacological baroreflex test (Gerlach et al., 2019). Based on their findings they suggest the activation of the NTS, the RVLM and/or raphe nuclei during these physiological tests. During the pharmacologically baroreflex test changes in BOLD signal were identified in several brainstem nuclei, including the NTS, RVLM, CVLM and nucleus raphe obscurus (Gerlach et al., 2019). Our finding of increased BOLD responses in the dorsal medulla during the CPT are in line with these previous studies using other physiological tests.

Most of the studies that have reported BOLD signal changes in the brainstem region used the same normalization for the brainstem and cerebellum as for the cortical regions. As subcortical structures are a lot smaller, some with cross-sectional diameters of only a few millimeters, the inter-subject alignment of these small regions is suboptimal and less accurate when using the same normalization as for the larger cortical structures (Sclocco et al., 2018). In addition, those studies are conducted using a field strength of three Tesla or lower where achieving an adequate signal-to-noise ratio for voxels in the subcortical regions requires a spatial resolution of 2–4 mm and a 2-3-fold smoothing kernel during preprocessing. Thus, they are working at the spatial resolution limit of 3T for brainstem imaging with standard acquisition sequences (Beissner, 2015).

Functional imaging of the brainstem is highly sensitive to physiological noise; i.e., cardiorespiratory noise, but also chest motion, pulsatile effects due to the cardiac and respiratory pulse pressure waves in adjacent arteries, cerebrospinal fluid spaces and the parenchyma, which influence the MRI signal in the small nuclei of the brainstem in close proximity of blood vessels. Moreover, the brainstem is located closer to the heart and lungs and hence BOLD signal in the brainstem nuclei is more affected by these noise sources than the cortical structures (Sclocco et al., 2018). Not all of the previous studies considered the high susceptibility of the brainstem nuclei for cardiac and respiratory noise. However, those who recorded respiratory and/or heart rates did not use them to correct for the physiological noise. Thus, previous results for the brainstem region might have been biased because of these confounders.

In our study, the activation of the medulla was detected only with a brainstem-specific analysis with the SUIT toolbox combined with correction for several cardiac and respiratory noise parameters. The SUIT toolbox uses a brainstem and cerebellum mask for the analysis of the functional images as well as a high resolution atlas template of the brainstem and cerebellum in the normalization (Diedrichsen, 2006). By combining ultra-high field MRI (7 Tesla), a specific toolbox (SUIT toolbox) and physiological noise corrections (Glover et al., 2000; Reynaud et al., 2017), we increased the precision of both the acquisition and the analysis of the functional images of the brainstem regions.

Despite the encouraging results showing that a low grade CPT increases BOLD signal in the medulla, this study has still several limitations. The first is the intermittent non-invasive BP measurement outside the scanner. BP measurement can be improved by continuously recording it. However, for now, it is not possible to measure BP inside the 7T MRI scanner, because none of the devices currently available are compatible with a 7T scanner.

Secondly, the lower thermal conductivity of the gel packs might have induced smaller increases in BP and smaller BOLD responses in the specific brain and brainstem regions. Foot immersion in icy water is, however, difficult to perform during MRI studies as it involves movement of the legs and hence creates movement artifacts and confounded activations in brain regions associated with the leg movement. Future studies should test an MRI-compatible device that can reproduce the conditions of the original CPT involving the full immersion of the feet in icy water without feet and leg movements.

Thirdly, cortical regions showing an increased CPT-associated BOLD signal are also involved in pain sensation. Due to the lack of pain assessment or evaluation in our study, we cannot exclude that a part of the BOLD response was due to pain instead of cold stress. However, no pain sensation was reported by the participants. Further research should include some form of standardized pain measurement or stimuli.

Although ultra-high field BOLD fMRI was used in this study, the voxel size might still be too large for a more accurate measurement of BOLD responses in the brainstem. In addition, because of the higher susceptibility of the brainstem to physiological noise it might also be better to scan for shorter durations or shorter stimulation periods or perform a small acquisition focusing only in subcortical regions. The limited statistical significance we obtained might be due to the small number of subjects as well as variations in the hemodynamic responses, which were found to be slower in some regions.

## Conclusion/Future Perspectives

The modified CPT induced detectable BOLD intensity changes in the medulla, as well as other brain regions such as the supplementary motor cortex of the lower body, the midcingulate and the right insula. A brainstem-specific analysis had to be used in order to detect the BOLD signal intensity changes in the dorsal medulla of the brainstem encompassing the NTS and the dorsal motor nucleus of the vagus nerve. Our results in healthy participants are encouraging for future research studies focusing on specific populations such as hypertensive patients, which have an increased sympathetic drive. Moreover, BOLD fMRI during a CPT might be a potential tool to detect differences in brain signal intensity changes in hypertensive patients compared to healthy subjects, and might provide new insights into the pathophysiological mechanisms of blood pressure regulation in hypertension patients.

## Data Availability Statement

The datasets generated for this study are available on request to the corresponding author.

## Ethics Statement

The studies involving human participants were reviewed and approved by the Commission Cantonale d’Éthique de la Recherche sur l’Être Humain (Canton of Vaud, Switzerland). The participants provided their written informed consent to participate in this study.

## Author Contributions

MH-B conducted the experiments, acquired the data, analyzed the data, and wrote the manuscript. FM, LP, OR, and DB conducted the experiments. OR and DB designed the protocols for MRI acquisition and acquired the MRI data. SD analyzed the data and revised the manuscript. DV and GW designed the research study, interpreted the results and revised the manuscript.

## Conflict of Interest

The authors declare that the research was conducted in the absence of any commercial or financial relationships that could be construed as a potential conflict of interest.

## Footnotes

1 www.fil.ion.ucl.ac.uk/spm

2 http://www.alivelearn.net/xjview

## Abbreviations

BOLD: Blood oxygen-level dependent
BP: blood pressure
CPT: cold pressor test
CVLM: caudal ventrolateral medulla
fMRI: functional magnetic resonance imaging
NTS: nucleus of the solitary tract
ROI: region of interest
RVLM: rostral ventrolateral medulla
SPM: statistical parametric mapping
SUIT: spatially unbiased infra-tentorial template
