# Supplementary material for: Brainstem Correlates of a Cold Pressor Test Measured by Ultra-High Field fMRI
Source: Front Neurosci. 2020 Jan 31;14:39. doi: 10.3389/fnins.2020.00039 (PMC7005099; doi:10.3389/fnins.2020.00039)
Supplement: Supplementary file 1 [file Data_Sheet_1.PDF]

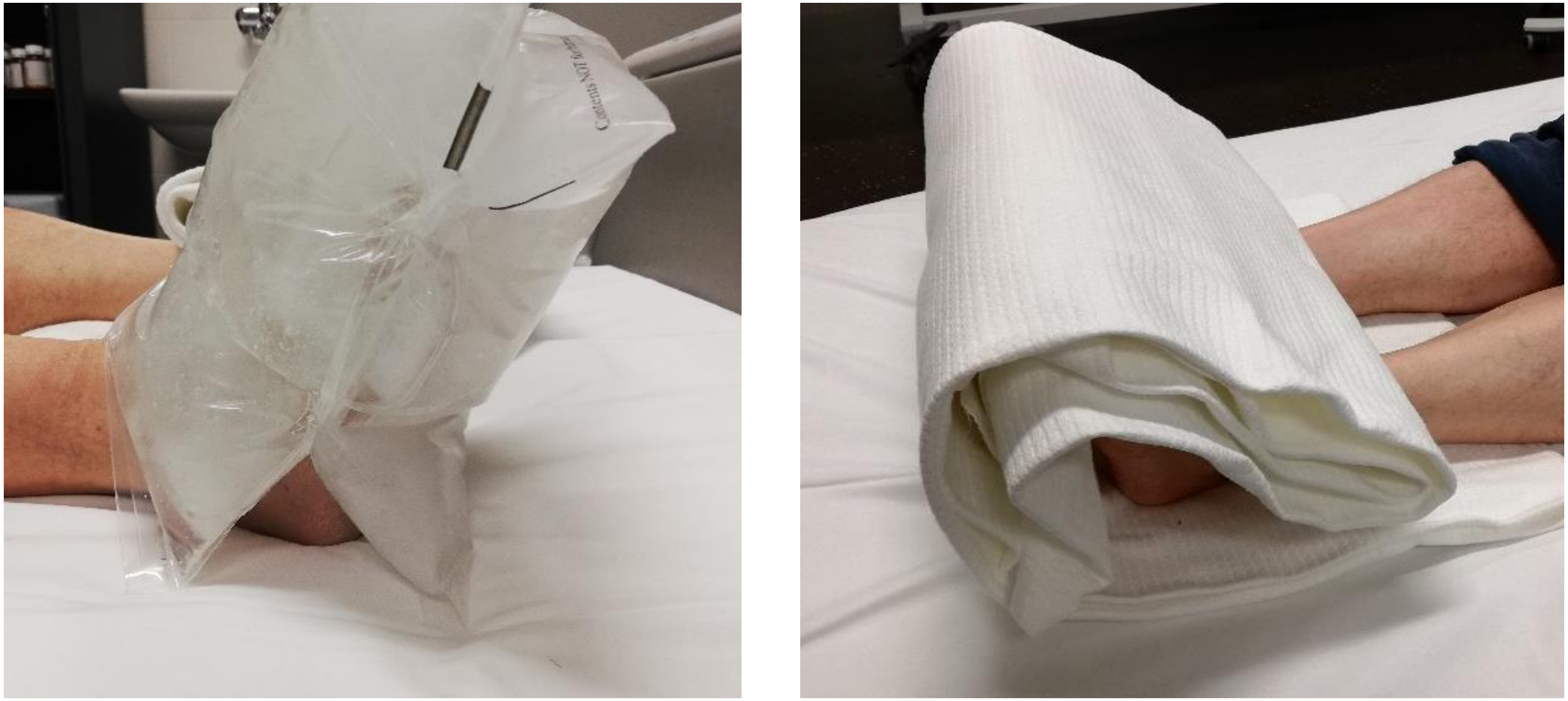

Supplementary figure 1. Application of the modified cold pressor test using the ice-cold gel packs wrapped around the feet (left) and the control test with the blanket (right).
