# Supplementary material for: Brainstem Correlates of a Cold Pressor Test Measured by Ultra-High Field fMRI
Source: Front Neurosci. 2020 Jan 31;14:39. doi: 10.3389/fnins.2020.00039 (PMC7005099; doi:10.3389/fnins.2020.00039)
Supplement: Supplementary file 2 [file Data_Sheet_2.PDF]

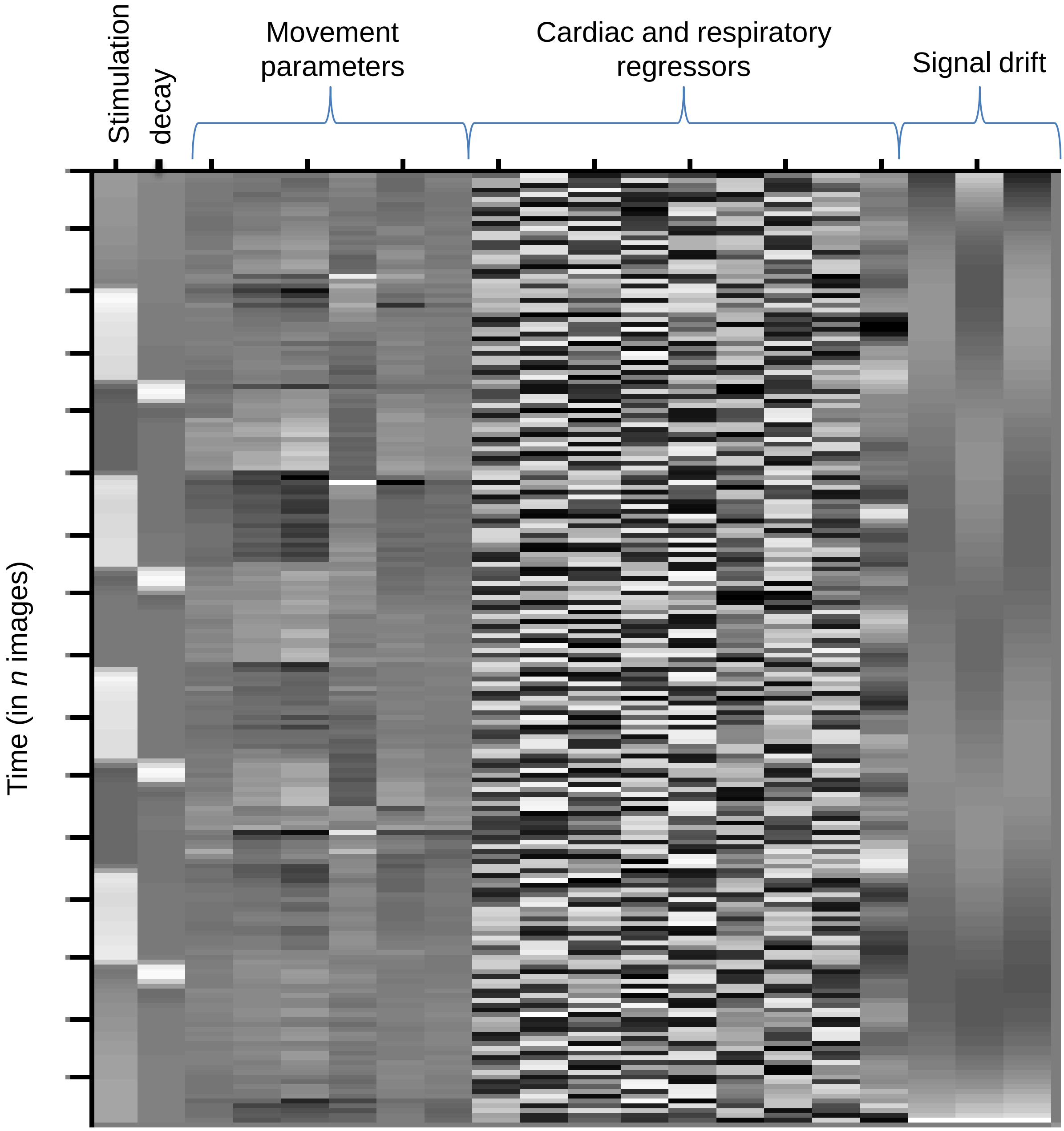

Supplementary figure 2. The general linear model used for the CPT stimulus showing the stimulus, decay, the six movement parameters, the cardiac and respiratory regressors and the signal drift as function of time (in 314 images).
