# Supplementary material for: Brainstem Correlates of a Cold Pressor Test Measured by Ultra-High Field fMRI
Source: Front Neurosci. 2020 Jan 31;14:39. doi: 10.3389/fnins.2020.00039 (PMC7005099; doi:10.3389/fnins.2020.00039)
Supplement: Supplementary file 3 [file Data_Sheet_3.pdf]

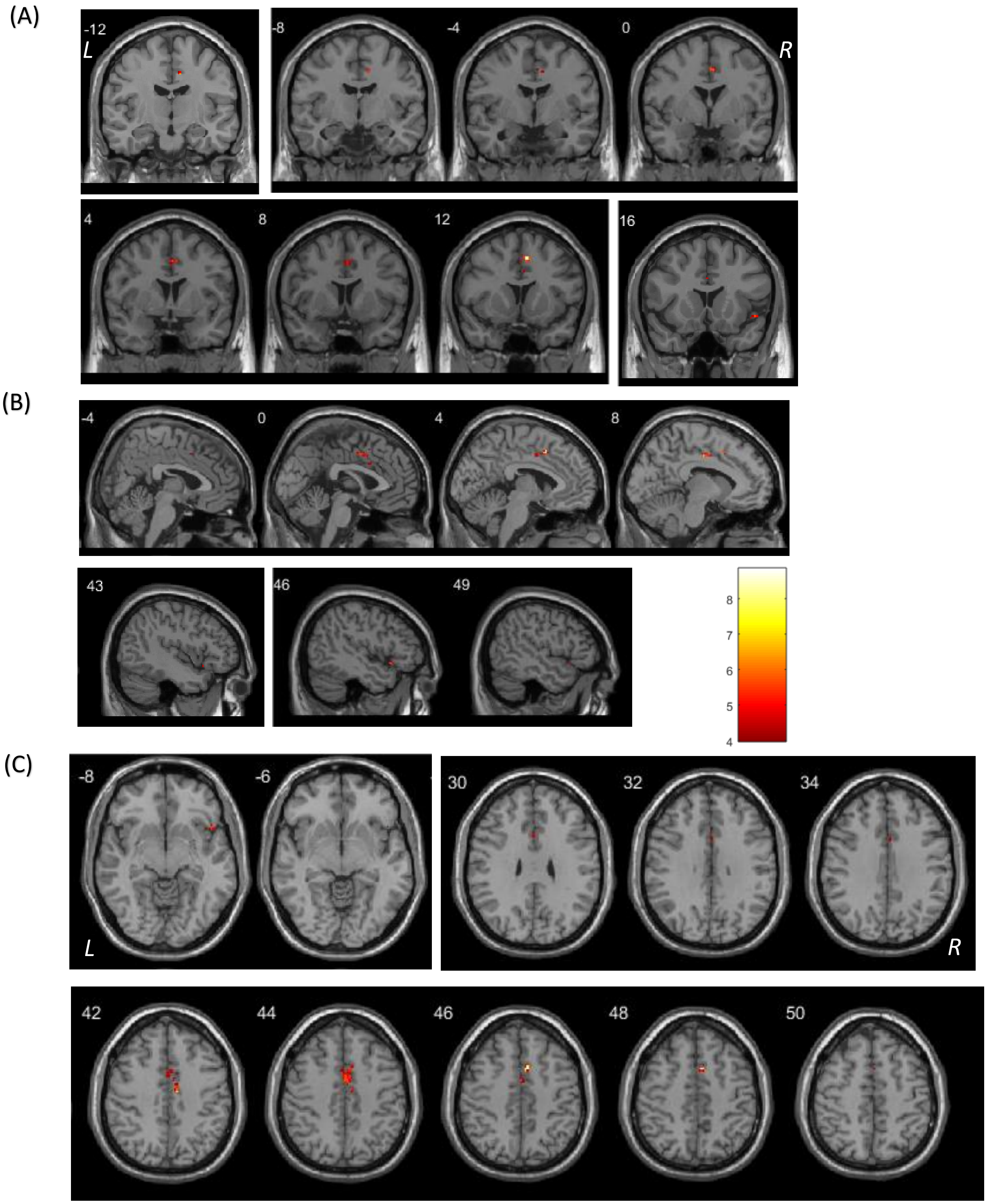

Supplementary figure 3. Significant activated clusters resulting from the whole brain analysis with the contrast CPT vs. control at group level (n=11; threshold of 4.14 for p<0.001 (uncorrected), cluster threshold of 6) overlaid onto the T1 reference image of SPM12. Data was corrected for motion, physiological noise and scanner drift. A: coronal images, B: sagittal images, C: axial images. *L* Left, *R* Right.
